# Cancer-associated thrombosis: prevention and treatment

**DOI:** 10.3747/co.2008.177

**Published:** 2008-01

**Authors:** K.M.J. Brose, A.Y.Y. Lee

**Affiliations:** * Queen’s University, Kingston, Ontario; † Department of Medicine, McMaster University, Hamilton, Ontario

**Keywords:** Neoplasm, deep vein thrombosis, thromboembolism, prophylaxis, low molecular weight heparin, heparin, warfarin

## Abstract

Patients with cancer are at high risk to develop venous thromboembolism, and they are also more likely to develop complications from anticoagulant treatment. Because little research has focused on the oncology population to date, the optimal methods of prophylaxis and treatment remain uncertain in some clinical situations. Currently, low molecular weight heparin and warfarin are the most frequently used pharmacologic agents; however, they have their limitations. Other therapeutic options, such as inferior caval filters, are poorly studied and remain controversial. A summary of the most recent evidence on the prevention and treatment of venous thromboembolism in cancer patients is presented here.

## 1. INTRODUCTION

It is well established that cancer patients are at an increased risk of venous thromboembolism (vte). In fact, the presence of malignancy increases the risk of vte by a factor of 4 to 6, and large population-based studies show that the incidence of vte is on the rise 1,2. Overall, cancer patients constitute 15%–20% of the patients diagnosed with vte, and depending on the type of tumour, extent of malignancy, type of cancer treatment, and presence of other risk factors, 1%–25% of patients with malignancy will develop thrombosis.

Furthermore, cancer-associated thrombosis is linked with poor prognosis, and it is the second leading cause of death in cancer patients 3. In one study of a population registry, the 1-year survival of cancer patients diagnosed with vte was one third that of cancer patients without vte (12% vs. 36%) matched for sex, age, tumour type, and duration of the cancer 4. In another population-based study, the in-hospital mortality for cancer patients who developed vte while in hospital was double that for patients who did not develop the complication 5. This increase in mortality is partly attributable to fatal pulmonary embolism, but it also reflects the advanced stages of cancer and underlying aggressive tumour biology in these patients.

Cancer patients are also prone to adverse effects and failure of anticoagulant therapy. In comparison with patients without cancer, patients with cancer who are receiving warfarin experience 2 to 6 times more major bleeding episodes and 2 to 3 times more vte recurrences 6,7. Based on prospective studies, the annual risk of recurrent vte is 21%–27% and the annual risk of major bleeding is 12%–13%.

Primary prevention is the most effective way to reduce the morbidity and mortality associated with cancer-associated thrombosis, but surveys and registries consistently report the failure of physicians to comply with established guidelines leading to underutilization of primary prophylaxis in oncology patients 8. Given the impact that vte has on this high-risk population, an increased focus on identifying optimal methods to prevent and treat vte in patients with malignancy is needed. Current management strategies, and the evidence supporting them, are presented here.

## 2. PRIMARY PREVENTION: SURGICAL SETTINGS

The risk of vte in cancer patients undergoing surgery has been estimated to be as high as 50% without prophylaxis 9. The exact risk varies with the type of surgery, but anticoagulants are generally effective in reducing the risk by 50%–80%. Tables I to iii summarize the results from randomized controlled trials evaluating the efficacy and safety of anticoagulant prophylaxis in various oncologic surgery settings 10–23.

### 2.1 Major Abdominal and Pelvic Surgery

After major abdominal or pelvic cancer surgery, pharmacologic intervention with anticoagulants for 7–14 days postoperatively can reduce the risk of vte to 1.3% for symptomatic deep vein thrombosis (dvt) and 0.4% for fatal pulmonary embolism. However, these numbers still represent a doubling or tripling of the risk as compared with patients without malignancy 24,25. Of the agents available for thromboprophylaxis, low molecular weight heparins (lmwhs) provide the most convenient, efficacious, and safe option (Table I) 10–14,26. Compared with lmwhs, unfractionated heparin requires 3-times-daily injection and has a higher risk of heparin-induced thrombocytopenia. And although fondaparinux appears to be comparable to lmwh in efficacy and safety, limited data are available for it in the cancer setting, and a specific antidote for it is lacking. A *post hoc* subgroup analysis of cancer patients in a randomized trial found a lower risk of vte with fondaparinux than with dalteparin, but that finding requires confirmation in future studies 12.

However, as hospital stays have shortened, the duration of prophylaxis may be inadequate for some patients. Cancer patients are at particularly high risk, considering that many of them undergo extensive surgery and require prolonged periods of recovery. In a prospective cohort study in which 2373 cancer patients were followed for a minimum of 30 days after surgery, 40% of symptomatic VTE occurred more than 3 weeks postoperatively, and 46% of deaths were the result of fatal pulmonary embolism 27. Clinical factors found to be associated with a higher risk of vte were previous history of vte [odds ratio (or): 6.0], anaesthesia lasting 2 hours or longer (or: 4.5), bed rest for 4 days or longer (or: 4.4), an advanced stage of cancer (or: 2.7), and age 60 years or older (or: 2.6).

Randomized controlled trials have shown that continuing prophylaxis with a lmwh up to 30 days postoperatively can reduce the risk of vte by 60% without increasing the risk of bleeding (Table I) 13,14. Based on these and other supportive studies, it is reasonable to prescribe extended prophylaxis in patients with one or more risk factors for vte.

### 2.2 Neurosurgery

Neurosurgical patients present a challenging prophylaxis problem because of the underlying concern of intracerebral hemorrhage combined with a high incidence of vte. In fact, craniotomy for brain neoplasm carries a vte risk of 60% in the postoperative period and 23% at 1 year 28. This risk can be reduced by 40% with the use of lmwh prophylaxis starting 24 hours postoperatively (Table II) 15–18. The risk of major bleeding, including intracranial hemorrhage, is less than 3%, but that risk is increased if lmwh prophylaxis is administered preoperatively 29. However, given the devastating consequences of an intracranial hemorrhage, most neurosurgeons prefer to use mechanical compression devices in the early operative period and to start pharmacologic prophylaxis when patients are more stable.

### 2.4 Gynecologic Surgery

Compared with the previous two groups, women undergoing gynecologic cancer surgery have a lower risk of vte—about 2%. But that level of risk represents an increase by a factor of 5 over the risk in women having surgery for benign gynecologic problems 9. As with abdominal and pelvic surgery, unfractionated heparin and lmwh are both equally effective and safe in preventing dvt in this setting ) (Table III 19–23,30. Mechanical devices have also been shown to be effective.

### 2.5 Other Surgeries

There is a paucity of research on the risk of vte and on its prevention in other surgical settings. Based on limited data and extrapolation from other surgical groups, unfractionated heparin and lmwh both appear relatively safe and effective, but solid evidence is lacking.

### 2.6 Mechanical Prophylaxis in Surgical Settings

As shown in one meta-analysis 31, compression stockings are effective in reducing the risk of vte by two thirds in patients at moderate risk. Pneumatic compression devices are also effective, but they are cumbersome and interfere with mobilization. Also, they are more likely than pharmacologic prophylaxis to fail in high-risk groups 32. Compression devices are therefore currently reserved for situations in which anticoagulation is contraindicated. These devices are most effective when applied intraoperatively or immediately after surgery (Table IV)^33^–^35^. Whether mechanical methods in combination with anticoagulants result in additional risk reduction is not clear.

### 2.7 Summary: Surgical Settings

Good evidence supports the routine use of postoperative thromboprophylaxis in patients undergoing surgery for cancer (Tables I to IV). That finding is endorsed by international and national consensus guidelines, including those from the American College of Chest Physicians (accp)^9^, the American Society of Clinical Oncology (asco)^36^, and the Italian Association of Medical Oncology (aiom)^37^. Unfractionated heparin and lmwh are equally effective in preventing vte and have a comparable risk for bleeding. Less experience and data are available for fondaparinux. Extended prophylaxis should be strongly considered in patients with additional risk factors for vte. In those patients who have a contraindication for anticoagulation, mechanical methods are reasonable substitutes.

## 3. PRIMARY PREVENTION: MEDICAL SETTINGS

### 3.1 Ambulatory Patients

The risk of symptomatic vte in the ambulatory outpatient setting is lower than that in the surgical setting, and little research had been done to investigate the role of anticoagulant prophylaxis. More recently, three separate randomized controlled trials have evaluated lmwh prophylaxis in patients with metastatic breast cancer, non-small-cell lung cancer, and grades and iii and iv malignant glioma (Table V) 38–40. No significant difference in vte or major bleeding was observed between the groups receiving lmwh and placebo. Consequently, routine prophylaxis is not recommended in ambulatory patients. Notably, the risk of vte and major bleeding appear to vary considerably, depending on the type of tumour.

On the other hand, as many as 20%–30% of patients receiving thalidomide in combination with chemotherapy or high-dose corticosteroids for treatment of multiple myeloma will develop symptomatic vte 41,42. Although many studies have reported the use of warfarin, aspirin, or lmwh, insufficient reliable data are available to support the efficacy and safety of these agents. Whether lenalidomide is also associated with a high risk of vte is uncertain. Patients receiving vascular endothelial growth factor inhibitors such as bevacizumab also have an increased risk of arterial and, possibly, venous thrombosis 43,44, but because of an increased bleeding tendency already associated with this treatment, anticoagulation prophylaxis is not recommended.

### 3.2 Hospitalized Patients

No study has been conducted in cancer patients to determine the effects of vte prophylaxis in the inpatient setting. Large randomized trials that included small numbers of cancer patients have shown that lmwh or fondaparinux can reduce the vte risk by 50% or more, but whether such results can apply to all hospitalized cancer patients is questionable, because of the higher risks for vte and major bleeding in those patients 45–47.

### 3.3 Summary: Medical Settings

Based on limited data, routine prophylaxis in the outpatient setting is not recommended, but prophylaxis should be considered in patients receiving thalidomide- or lenalidomide-containing regimens. Anticoagulant prophylaxis for hospitalized patients is warranted based on studies in noncancer patients, but the risk–benefit ratio is uncertain, given the lack of randomized trial evidence.

## 4. PROPHYLAXIS FOR CENTRAL VENOUS CATHETERS

Central venous catheters are frequently placed in cancer patients, and those devices represent an ongoing thrombogenic focus 48. Many attempts have been made to reduce the incidence of thrombotic complications in this setting, but randomized placebo-controlled trials have failed to show a reduction in catheter-related thrombosis with thromboprophylaxis. In particular, low-dose warfarin 49, adjusted-dose warfarin 50, and prophylactic-dose lmwh 51,52 do not reduce the 4% risk of symptomatic catheter–related thrombosis (Table VI) 49–52. For that reason and because of the possibility of increased risk of bleeding, the routine use of prophylaxis in this setting is not recommended 9.

## 5. SECONDARY PREVENTION

### 5.1 Treatment of VTE

Traditional therapy for acute vte has consisted of initial therapy with unfractionated heparin or lmwh, followed by long-term warfarin therapy. Unfortunately, many cancer patients tolerate warfarin poorly, especially when receiving chemotherapy, with its ensuing gastrointestinal and hematologic side effects. Also, despite maintenance of therapeutic levels of warfarin, one third of patients experience recurrent vte or treatment-related bleeding 6,7.

However, lmwh is clearly superior to warfarin with respect to convenience and efficacy. Given as a once-daily subcutaneous injection, lmwh does not require routine laboratory monitoring, has few drug interactions, and does not rely on gastrointestinal absorption. In the clot study, 676 cancer patients with acute dvt or pulmonary embolism were randomized to a 6-month course of traditional therapy with dalteparin followed by warfarin, or to dalteparin alone 53. To reduce the risk of bleeding in the dalteparin-only group, the dalteparin dose was reduced by 20%–25% after the first month of therapy. After 6 months of treatment, the long-term dalteparin group experienced a 52% reduction in symptomatic recurrent vte (17% vs. 9%, *p* = 0.002) as compared with the group continuing on warfarin. In other words, 1 episode of recurrent vte was prevented for every 13 patients treated. Furthermore, the groups showed no significant difference in bleeding (4% vs. 6% respectively) and no difference in overall mortality.

Based on those results and on supportive findings from other trials (Table VII) 53–56, the 2004 accp guidelines 57, the asco vte guidelines 36, and aiom 37 recommend the use of lmwh alone for treatment of vte in most patients with cancer. Currently, only dalteparin has received regulatory approval for long-term treatment of symptomatic vte in patients with cancer.

### 5.2 Recurrent VTE

As mentioned earlier, recurrence of vte is more common in the setting of warfarin therapy than of lmwh therapy. Unfortunately, the optimal treatment for such patients has yet to be determined. Inferior caval filters have frequently been used in cases of warfarin failure, but evidence from a large randomized trial primarily in patients without cancer showed that, although the risk of short-term pulmonary embolism is reduced by about three quarters, patients receiving a filter are about twice as likely to develop recurrent vte. Also, no difference in overall survival was seen 58. Furthermore, because of the increased risk of recurrent vte, cancer patients may be at higher risk for development of postphlebitic syndrome.

Based on the foregoing findings, using caval filters as treatment for recurrent vte cannot be recommended. Rather, changing a warfarin regimen to lmwh, or increasing the dose of lmwh would be appropriate.

### 5.3 Duration of Therapy

The optimum duration of anticoagulation in patients with cancer has not been investigated. Extrapolating from populations without cancer, most patients should receive a minimum of 3–6 months of therapy. However, given that cancer represents an ongoing risk factor in this population, the general recommendation is to continue anticoagulation for long as evidence of active malignancy continues. It is of foremost importance that patient care be tailored to suit the individual, with due consideration for quality of life and life expectancy.

### 5.4 Summary of Secondary Prevention

First-line treatment of vte in patients with cancer is lmwh for a minimum of 3–6 months. This approach is endorsed by the accp, asco, aiom, and the National Comprehensive Cancer Network. The optimum duration is not known, but treatment is usually continued for as long as evidence of malignancy continues.

## 6. FUTURE DIRECTIONS

The lmwhs have simplified and improved the prevention and treatment of vte in patients with cancer. Much work must still be done to help identify high-risk patients who would benefit from primary thromboprophylaxis for extended periods after cancer surgery, during medical hospitalization, for prevention of catheter-related thrombosis, and for prevention of vte associated with highly thrombogenic agents (thalidomide, for example). Also, optimal management in patients with established vte should be investigated, especially with regard to duration of therapy and the potential role, if any, of invasive strategies such as filter insertion. Lastly, education of physicians to improve the appropriate utilization of prophylaxis in various settings will go a long way toward reducing morbidity and mortality in this vulnerable population.

## 7. SUMMARY

Recommendations for managing risk of thrombotic events:

- Cancer patients undergoing surgery require vte prophylaxis, and this prophylaxis can safely be extended in high-risk patients after discharge.
- Medical inpatients with cancer should receive vte prophylaxis unless absolute contraindications—active bleeding, for instance—are present.
- Prophylaxis for central venous catheters is not recommended.
- Prophylaxis in ambulatory patients is not recommended, except in those who are receiving thalidomide- or lenalidomide-based combination chemotherapy.
- Venous thromboembolism is best treated with lmwh for a minimum of 3–6 months, and treatment can be continued for as long as active cancer is present.
- Inferior caval filters prevent pulmonary embolism in the short term, but they carry a long-term risk of increased vte and should therefore be avoided when anticoagulation can be administered.

## Figures and Tables

**TABLE I:** Randomized controlled trials for primary prophylaxis in cancer-related major abdominal and pelvic surgery

Trial	Surgical setting	Cancer patients (*n*)	Regimen		Duration of treatment	Outcome	Incidences					
			Study	Control			vte			Major bleeding		
							Study (%)	Control (%)	*p* Value	Study (%)	Control (%)	*p* Value
Enoxacan Study Group, 1997 10	Abdominal and pelvic	631	Enoxapari 40 mg daily	ufh 5000 IU 3 times daily	10 Days	vte on bilateral venography or pulmonary scintigraphy	14.7	18.2	>0.05	4.1	2.9	>0.05
McLeod *et al.*, 2001 11	Colorectal	324	Enoxaparin 40 mg daily	ufh 5000 IU 3 times daily	Up to 10 days	pe, venographic dvt at postoperative days 5, 9	13.9	16.9	0.052	2.7^a^	1.5^a^	0.136^a^
Agnelli *et al.*, 2005 12	Major abdominal	1941	Fondaparinux 2.5 mg daily	Dalteparin 5000 IU daily	5–9 Days	Venographic dvt or pe up to postoperative day 10	4.7	7.7	<0.05	3.4	2.5	0.355
Bergqvist *et al.*, 2002 13	Abdominal and pelvic	332	Enoxaparin 40 mg daily	Enoxaparin 40 mg daily	27–31 Days (study) 6–10 Days (control)	Bilateral venography between days 25 and 31	4.8	12.0	0.02	0.4	0	>0.99
Rasmussen *et al.*, 2006 14	Major abdominal	198	Dalteparin 5000 IU daily	Dalteparin 5000 IU daily	28 Days (study) 7 Days (control)	Venographic vte on postoperative days 7–28	8.8	19.6	0.03	0.5^a^	1.8^a^	>0.05^a^

a Results for the overall study population, including cancer and noncancer patients.

vte = venous thromboembolism; ufh = unfractionated heparin; pe = pulmonary embolism; dvt = deep vein thrombosis.

**TABLE II:** Randomized controlled trials for primary prophylaxis in cancer-related neurosurgery

Trial	Surgical setting	Patients (*n*)	Regimen		Duration of treatment	Outcome	Incidences					
			Study	Control			vte			Major bleeding		
							Study (%)	Control (%)	*p* Value	Study (%)	Control (%)	*p* Value
Nurmohamed *et al.*, 1996 15	Craniotomy or spinal surgery for tumour or injury	485	Nadroparin 7500 IU daily and gcs	Placebo and gcs	10 Days or until discharge	Venographic dvt at day 10	18.7	26.3	0.047	2.50	0.80	0.87
Agnelli *et al.*, 1998 16	Elective cranial or spinal surgery for tumours	307	Enoxaparin 40 mg daily and gcs	Placebo and gcs	At least 7 days	Venographic dvt at day 8, or pe	17	33	0.004	3	3	>0.05
Goldhaber *et al.*, 2002 17	Craniotomy for brain tumour	150	Enoxaparin 40 mg daily and gcs and ipc	ufh 5000 IU twice daily and gcs and ipc	Until vte or discharge	Ultrasonographic dvt at discharge	12	6.7	0.401	2.7	1.3	>0.05
Macdonald *et al.*, 2003 18	Craniotomy	100	Dalteparin 2500 IU daily and ipc	ufh 5000 IU twice daily and ipc	7 Days, or until ambulating	pe, or ultrasonographic dvt at 1 month	4	0	>0.05	4	2	>0.05

vte = venous thromboembolism; gcs = graduated compression stockings; dvt = deep vein thrombosis; pe = pulmonary embolism; ipc = intermittent pneumatic compression; ufh = unfractionated heparin.

**TABLE III:** Randomized controlled trials for primary prophylaxis in cancer-related gynecologic surgery

Trial	Patients (*n*)	Regimen		Duration of treatment	Outcome	Incidences					
		Study	Control			vte			Major bleeding		
						Study(%)	Control (%)	*p* Value	Study (%)	Control (%)	*p* Value
Heilmann *et al.*, 1989 19	300	lmwh 1500 IU daily	ufh 5000 IU 3 times daily	7 Days	pe, dvt by impedance plethysmography to day 7	1.3	4.0	>0.05		No significant difference in clinical and laboratory measures, specific incidence of major bleeding not cited	
Clarke–Pearson *et al.*, 1990 20	200	ufh 5000 IU every 8 hours pre- and postoperatively	No prophylaxis	Until discharge	pe, dvt by ^125^I-labelled fibrinogen scan, impedance plethysmography to day 30	6.2	18.4	0.008		No significant difference in clinical and laboratory measures, specific incidence of major bleeding not cited	
Fricker *et al.*, 1988 21	80	Dalteparin 2500 IU 2 hours preoperatively and at 12 hours, then 5000 IU daily	ufh 5000 IU 2 hours preoperatively, then 3 times daily	10 Days	pe, dvt by ^125^I-labelled fibrinogen scan, venography to 4 weeks	0	2.5	>0.05	5.1	2.5	>0.05
Von Tempelhoff *et al.*, 1997 22	60	lmwh 3000 IU daily	ufh 5000 IU 3 times daily	7 Days	pe on scintigraphy, by impedance dvt plethysmography, venography, up to day 30	6.7	0	>0.05		Not assessed	
Heilmann *et al.*, 1998 23	324	Certoparin 3000 IU daily	ufh 5000 IU 3 times daily	7 Days	dvt by impedance plethysmography, venography up to day 10	6.3	6.1	1.0	16.8	28.7	0.01

vte = venous thromboembolism; lmwh = low molecular weight heparin; ufh = unfractionated heparin; pe = pulmonary embolism; dvt = deep vein thrombosis.

**TABLE IV:** Randomized controlled trials for primary prophylaxis using pneumatic compression stockings

Trial	Surgical setting	Patients(*n*)	Regimen		Duration of treatment	Outcome	Incidence of vte		
			Study	Control			Study (%)	Control (%)	*p* Value
Clarke–Pearson *et al.*, 1993 33	Gynecologic malignancy	208	ufh 5000 IU 3 times daily pre- and postoperatively	Intra- and postoperative ipc	ufh: 7 days postoperatively, or until discharge ipc: 5 days postoperatively, or until discharge	dvt by ^125^I-labelled fibrinogen scan, impedance plethysmography; pe on scintigraphy up to day 30 postoperatively	6.5	4.0	0.54
Clarke–Pearson *et al.*, 1984 34	Gynecologic malignancy	194	Intra- and postoperative ipc	No prophylaxis	Maximum 24 hours postoperatively	dvt by ^125^I-labelled fibrinogen scan, impedance plethysmography	18.6	12.4	>0.05
Clarke–Pearson *et al.*, 1984 35	Gynecologic malignancy	107	Intra- and postoperative ipc	No prophylaxis	5 Days	dvt by ^125^I-labelled fibrinogen scan, impedance plethysmography	12.7	34.6	<0.005

vte = venous thromboembolism; ufh = unfractionated heparin; ipc = intermittent pneumatic compression; dvt = deep vein thrombosis; pe = pulmonary embolism.

**TABLE V:** Randomized controlled trials for primary prophylaxis in medical outpatients with cancer

Trial	Medical setting	Patients(*n*)	Regimen		Duration of treatment	Outcome	Incidences					
			Study	Control			vte			Major bleeding		
							Study(%)	Control (%)	*p* Value	Study (%)	Control (%)	*p* Value
Levine *et al.*, 1994 38	Breast cancer stage iv	311	Warfarin 1 mg daily for 6 weeks, then adjusted for inr 1.3–1.9	Placebo	1 Week after completion of chemotherapy	Symptomatic vte	0.7	4.4	0.031	5.30	3.10	0.4
Haas *et al.*, 2005 39	Advanced breast cancer	353	Certoparin 3000 IU daily	Placebo	6 Months	Ultrasonographic dvt	4.0	3.9	>0.05	1.7	0	>0.05
	Advanced non-small-cell lung cancer	547	Certoparin 3000 IU daily	Placebo	6 Months	Ultrasonographic dvt	4.5	8.3	0.07	3.7	2.2	>0.05
Perry *et al.*, 2007 40	Glioma grade iii or iv	186	Dalteparin 5000 IU daily	Placebo	6 Months	pe, symptomatic dvt	11	17	0.3	5.1	1.2	0.2

vte = venous thromboembolism; dvt = deep vein thrombosis; pe = pulmonary embolism; inr = international normalized ratio.

**TABLE VI:** Randomized controlled trials for primary prophylaxis in central venous catheters in cancer patients

Trial	Patients (*n*)	Regimen		Duration of treatment	Outcome	Incidences					
		Study	Control			vte			Major bleeding		
						Study (%)	Control (%)	*p* Value	Study (%)	Control (%)	*p* Value
Couban *et al.*, 2005 49	255	Warfarin 1 mg	Placebo	Until dvt, or until line removed	cvc-associated thrombosis on ultrasound or venography	4.6	4.0	>0.05	0	2	0.5
Young *et al.*, 2005 50	1589	Adjusted warfarin for inr 1.5–2.0 or warfarin 1 mg	Placebo	Until dvt, or until line removed	cvc-associated thrombosis on ultrasound or venography	5	6	0.84	2	0.2	0.07
Verso *et al.*, 2005 51	321	Enoxaparin 40 mg daily	Placebo	Until dvt, or until line removed	Venographic dvt, or pe	14.1	18.0	0.35	0	0	>0.05
Karthaus *et al.*, 2006 52	425	Dalteparin 5000 IU daily	Placebo	12 Weeks	pe, venographic or ultrasonographic dvt	3.7	3.4	0.88	0.004	0	>0.05

vte = venous thromboembolism; dvt = deep vein thrombosis; cvc = central venous catheter; inr = international normalized ratio; pe = pulmonary embolism.

**TABLE VII:** Randomized controlled trials for treatment of cancer-related venous thromboembolism (vte)

Trial	Patients (*n*)	Regimen		Duration of treatment	Outcome	Incidences					
		Study	Control			vte			Major bleeding		
						Study (%)	Control (%)	*p* Value	Study (%)	Control (%)	*p* Value
Meyer *et al.*, 2002 54	146	Enoxaparin 1.5 mg/kg daily	Enoxaparin 1.5 mg/kg daily for 5–7 days, then warfarin at inr 2–3	3 Months	Combined recurrent vte and hemorrhage	10.5^a^	21.1^a^	0.09^a^	7.0	16.0	0.09
Lee *et al.*, 2003 53	672	Dalteparin 200 IU/kg daily for 1 month, then 150 IU/kg daily for 5 months	Dalteparin 200 IU/kg daily for 5–7 days, then warfarin at inr 2–3	As described in “Regimen”	Recurrent vte	9.0	17.0	0.002	6	4	0.27
Deitcher *et al.*, 2006 55	122	Enoxaparin 1 mg/kg twice daily, then 1.0 mg/kg daily or 1.5 mg/kg daily	Enoxaparin 1 mg/kg twice daily, then warfarin at inr 2–3	5 Days at twice daily, then 6 months	Recurrent vte	6.9 (1 mg), 6.3 (1.5 mg)	10.00	>0.05	6.5 (1 mg), 11.1 (1.5 mg)	2.90	>0.05
Hull *et al.*, 2006 56	200	Tinzaparin 175 IU/kg daily	ufh infusion then warfarin at inr 2–3	3 Months	Recurrent vte	6.0^b^	10.0^b^	>0.05^b^	7.0	7.0	>0.05

a Combined 3-month incidence of patients with either recurrent venous thromboembolism or major bleeding.

b 3-Month incidence of patients with recurrent venous thromboembolism.

vte = venous thromboembolism; inr = international normalized ratio.
